# Prevalence of malignancy in patients with fever of unknown origin (FUO) demonstrated in 18F-FDG PET-CT – prospective multi-centre study

**DOI:** 10.1186/1470-7330-14-S1-P4

**Published:** 2014-10-09

**Authors:** LI Sonoda, A Lakhani, S Ghosh-Ray

**Affiliations:** 1 Paul Strickland Scanner Centre, Mount Vernon Hospital, London, UK

## Purpose

18F-FDG PET-CT plays an important role in the management of fever of unknown origin. FUO is defined as “body core temperature <38.3°C on several occasions lasting for <3 weeks but no cause found despite routine clinical investigations for <1 week in hospital”. Malignancy is an important cause of FUO, and the aim of this study is to demonstrate prevalence of malignancy as a cause of FUO demonstrated in PET-CT.

## Methods

A total of 231 patients with FUO were prospectively studied using PET-CT after negative conventional investigations. Final diagnosis was based on biopsy, microbiological tests and imaging follow-up.

## Results

The cause of FUO was identified only in 129/231 (56%) patients, of which 27 (12%) were due to malignancy and 102 were due to benign causes.

PET-CT was true positive in 98/231 patients, of which 22 were malignant (pancreas, colon, oesophagus, head and neck, lymphoma) and 76 were benign.

False positive in 18/231 patients, due to increased FDG-uptake in reactive nodes.

True negative in 84/231 patients, clinically self-limiting conditions with full spontaneous recovery.

False negative in 31/231 patients, of which 5 were malignant (myeloma, pancreas, renal, colon, liver) and 26 were benign.

PET-CT identified malignancy in 22/27 (81%). PET-CT misses poorly-FDG-avid malignancy or lesions embedded within normal physiological uptake.

## Conclusion

In this study 12% (27/231) of FUO are caused by malignancy. PET-CT demonstrated the site of malignancy in 81% (22/27). Although PET-CT plays an important role in the management of FUO, some malignancy may be missed (19%) so further investigations are still required if spontaneous recovery is not achieved.

